# Reliability of light microscopy and a computer-assisted replica measurement technique for evaluating the fit of dental copings

**DOI:** 10.1590/1678-7757-2016-0590

**Published:** 2018-01-16

**Authors:** Heike Rudolph, Silke Ostertag, Michael Ostertag, Michael H. Walter, Ralph Gunnar LUTHARDT, Katharina Kuhn

**Affiliations:** 1 Universität Ulm, Zentrum für Zahn-, Mund- und Kieferheilkunde, Klinik für Zahnärztliche Prothetik, Ulm, Deutschland; 2 Technische Universität Dresden, Medizinische Fakultät Carl Gustav Carus, Poliklinik für Zahnärztliche Prothetik, Dresden, Deutschland; 3 Private practice, Esslingen, Deutschland; 4 Private practice, Stuttgart, Deutschland

**Keywords:** Prosthodontics, Computer-aided design, Computer-aided manufacturing, Permanent dental restoration, Reproducibility of results

## Abstract

The aim of this *in vitro* study was to assess the reliability of two measurement systems for evaluating the marginal and internal fit of dental copings.

**Material and Methods:**

Sixteen CAD/CAM titanium copings were produced for a prepared maxillary canine. To modify the CAD surface model using different parameters (data density; enlargement in different directions), varying fit was created. Five light-body silicone replicas representing the gap between the canine and the coping were made for each coping and for each measurement method: (1) light microscopy measurements (LMMs); and (2) computer-assisted measurements (CASMs) using an optical digitizing system. Two investigators independently measured the marginal and internal fit using both methods. The inter-rater reliability [intraclass correlation coefficient (ICC)] and agreement [Bland-Altman (bias) analyses]: mean of the differences (bias) between two measurements [the closer to zero the mean (bias) is, the higher the agreement between the two measurements] were calculated for several measurement points (marginal-distal, marginal-buccal, axial-buccal, incisal). For the LMM technique, one investigator repeated the measurements to determine repeatability (intra-rater reliability and agreement).

**Results:**

For inter-rater reliability, the ICC was 0.848-0.998 for LMMs and 0.945-0.999 for CASMs, depending on the measurement point. Bland-Altman bias was −15.7 to 3.5 μm for LMMs and −3.0 to 1.9 μm for CASMs. For LMMs, the marginal-distal and marginal-buccal measurement points showed the lowest ICC (0.848/0.978) and the highest bias (-15.7 μm/-7.6 μm). With the intra-rater reliability and agreement (repeatability) for LMMs, the ICC was 0.970-0.998 and bias was −1.3 to 2.3 μm.

**Conclusion:**

LMMs showed lower interrater reliability and agreement at the marginal measurement points than CASMs, which indicates a more subjective influence with LMMs at these measurement points. The values, however, were still clinically acceptable. LMMs showed very high intra-rater reliability and agreement for all measurement points, indicating high repeatability.

## Introduction

The fit of dental restorations has been subjected to numerous investigations. A poor marginal fit is associated with secondary caries^6^, which is among the most common causes of fixed partial-denture loss^28^. Both marginal and internal fit measurements are used to evaluate new materials and manufacturing procedures for dental restoration^9^ ^-^ ^13^ ^,^ ^21^ ^,^ ^26^.

Many measurement methods have been established to investigate the fit of dental restorations. To confirm a method's validity, however, two methods should be studied and compared^22^. A commonly used method is the internal silicone replica technique, described by Holmes, et al.^5^ (1989), which enables the investigation of both marginal and internal gaps. Light-body silicone replicas fill the space between the restoration and the die. They are coated from the inner or outer side with a heavy-body silicone of various colors. After stabilization of the thin light-body material, the replicas are cut in different planes for analysis by light microscopy^9^ ^,^ ^11^ ^-^ ^13^ ^,^ ^15^ ^,^ ^19^ ^,^ ^21^ ^,^ ^30^.

Another possibility for determining the replica's thickness is a computer-assisted technique that measures the optically captured replicas digitally^14^ ^,^ ^20^. The validity of the replica technique for the predictable reproduction of cement thickness, regardless of the measurement point location, has been proven^15^. However, evidence regarding the reliability and repeatability of the conventional light microscopy replica technique is sparse^21^, and none are available for the more recent computer-assisted replica technique. The reliability of a measurement method is determined by comparing the measurements performed by several investigators (inter-rater reliability and agreement), whereas repeatability is calculated by repeated measurements by the same investigator. Thus, repeatability can be referred to as intra-rater reliability and agreement, which is done throughout this paper.

This study focused on analyzing the reliability and repeatability of the conventional light microscopy replica technique by determining both inter-rater and intra-rater reliability and agreement for specific measurement point locations. As a second step, the study aimed to compare the conventional light microscopy replica technique to the more recent computer-assisted replica technique by means of the respective inter-rater reliability and agreement for specific measurement point locations.

The hypotheses for this *in vitro* study were that: (1) the conventional light microscopy replica technique for the analysis of dental coping fit shows high intrarater reliability and agreement; (2) the conventional light microscopy method and the computer-assisted replica technique for the analysis of dental coping fit show high inter-rater reliability and agreement; and (3) the intra-rater and inter-rater reliability and agreement are independent of the specific measurement point location for the conventional light microscopy technique (intra-rater and inter-rater) and the computer-assisted replica technique (inter-rater) for analyzing dental coping fit.

## Material and methods

### Manufacture of copings and replicas

**Figure 1:**
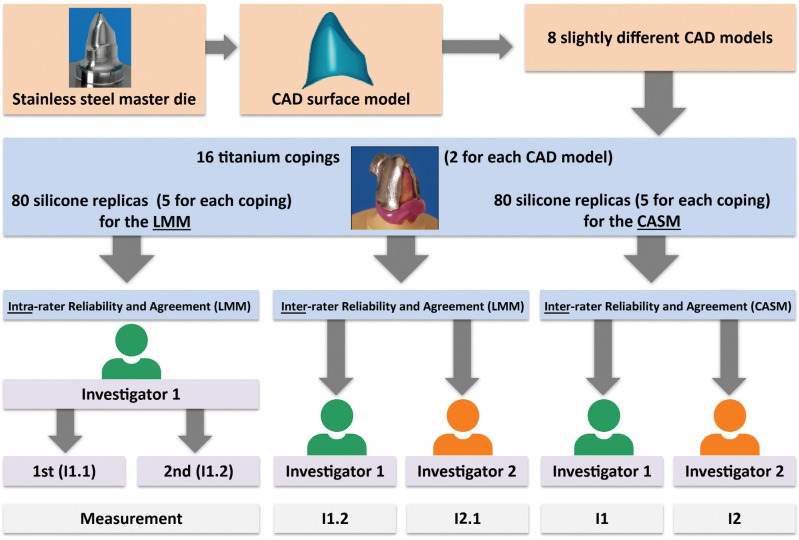
Study protocol; CAD= computer-aided design. LMM= light microscopy measurement; CASM= computer-assisted measurement; I1.1 and I1.2= first and second measurements of the first investigator (LMM); I2.1 = measurement of the second investigator (LMM); I1 and I2= measurements of the first and second investigators (CASM)

A prepared (chamfer) stainless steel maxillary canine (FDI 13) and its computer-aided design (CAD) surface model served as the master die (height 7.8 mm, cone angle 4°, bucco-oral diameter 10 mm at the margin). Using the CAD software (Surfacer^®^V. 10.0; Imageware Inc., Ann Arbor, MI, USA), the parameters were modified to create eight different CAD models resulting in varying fit. Therefore, the following parameters were modified: four CAD models showed high data density (point clouds of 123,029) and four CAD models showed low data density (point clouds of 8,513). Both data density groups were modified with regard to the fit by enlarging the originally sized data (1^st^ CAD model) in z-direction (height; 2^nd^ CAD model), in x-/y- direction (circumference; 3^rd^ CAD model) and x-/y-/z-direction (height and circumference, 4^th^ CAD model). Two titanium copings were manufactured for each of the eight different CAD models, resulting in 16 copings. For each titanium coping, five silicone replicas were produced for the light microscopy measurements (LMMs) and for the computer-assisted measurements (CASMs), respectively. This resulted in 80 (16×5) silicone replicas for the LMMs and 80 (16×5) silicone replicas for the CASMs, respectively (Figure 1).

While differences in fit influenced by different CAD model parameters were analyzed in a previous evaluation^14^, this study focused on the intra- and interrater reliability and agreement.

For the LMMs, the restoration inside was isolated by silicone oil (type 350; Caesar & Loretz, Hilden, Germany), which guaranteed that the replica would stay on the master die. The restoration was "cemented" with light-body addition-curing silicone (Dimension^®^ Garant L; 3MESPE AG, Seefeld, Germany) on the master die with a force of 20 N using a scale with a digital display (Leifheit AG, Nassau/Lahn, Germany). An individual marker ring with grooves fabricated with training alloy (Degussa Dental GmbH & Co., Hanau-Wolfgang, Germany) was used for subsequent reproducible segmentation. After 10 min, using pliers, the restoration was removed from the master die in the axial direction. The master die replicas were reinforced by coating them with a heavy-body addition-curing silicone (clear color contrast) before cutting.

The silicone replicas for the CASMs could not be produced on the master die due to its highly light- reflecting metallic surface, which was inappropriate for digitizing by the optical three-coordinate measurement system used (ODKM 97; Fraunhofer Institute for Applied Optics and Precision Engineering, IOF, Jena, Germany). Therefore, 80 plaster dies — one for each replica — were fabricated using the double mix impression technique. Using the plaster dies, replicas (Dimension^®^ Garant L; 3MESPE AG, Seefeld, Germany) were manufactured as described above for the LMMs. The light-body silicone replica was not coated with heavy-body silicone for stabilization because no cutting was involved.

All replicas for the LMMs and for the CASMs were manufactured by the same operator to guarantee comparability.

### LMMs

**Figure 2:**
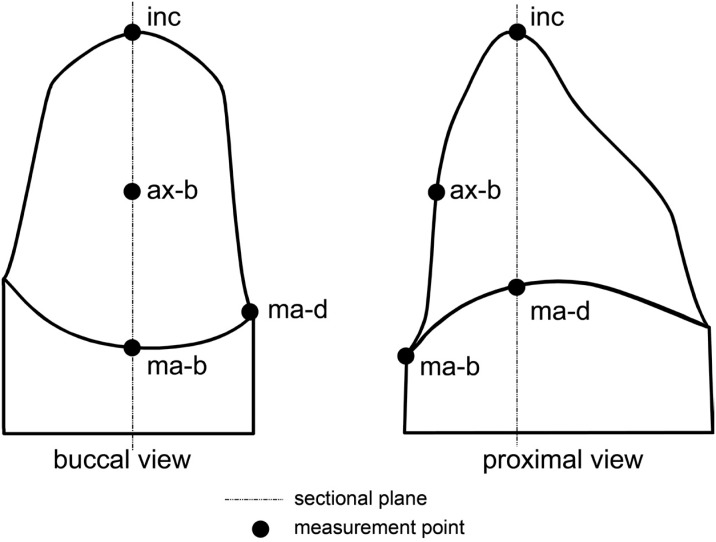
Measurement points: marginal-buccal (ma-b), marginal-distal (ma-d), axial-buccal (ax-b), incisal (inc); figure modified from Kuhn, et al.^14^ (2015) with permission of Elsevier

For the LMMs, the 80 coated replicas were cut with a scalpel in the mesio-distal and buccal-lingual directions according to the impressions of the marker ring. The method described by Holmes, et al.^5^ (1989) defined the "marginal gap" and the "internal gap". Two values were gained for the marginal-buccal (ma-b), marginal-distal (ma-d), and axial-buccal (ax-b) measurement points, and eight values were obtained for the incisal (inc) measurement point, as both sides of each sectional cut were considered (incisal: both sides of four sectional cuts) (Figure 2). At those measurement points, the replica's thickness was orthogonally determined using a measuring microscope (40x magnification; Zeiss, Jena, Germany). For all analyses, the mean values from both sides of each sectional cut were calculated, resulting in one value for each measurement point: ma-b, ma-d, ax-b, and inc. Thus, comparability with the computer-assisted measurements (see below) was obtained.

### CASMs

For the CASMs, the digitizing system ODKM 97 was used (measurement uncertainty of ~8 μm, as stated by the manufacturer (Fraunhofer Institute for Applied Optics and Precision Engineering, IOF, Jena, Germany)^25^. Each plaster die was digitalized once with a replica and once without a replica in the same spatial orientation. Thus, no further alignment of the two resulting point clouds was necessary^16^. Before digitization, the silicone replica was covered with Cerec^®^ powder (titanium oxide; VITA Zahnfabrik, Bad Säckingen, Germany). Calibration was performed each time the program was initiated and after every 10 measurements. While topical digitalization devices usually perform an automatic calibration before starting a measurement, older systems such as the ODKM 97 needed to be calibrated using standard geometries and a calibration software. The point clouds were processed using software tools (Argus; Fraunhofer Institute for Applied Optics and Precision Engineering, IOF, Jena, Germany) and aligned (Surfacer^®^ 10.6; Ann Arbor, MI, USA) with the corresponding CAD master model, as described by Luthardt, et al.^17^ (2003). Color- coded difference images (needle plot) in standard (ISO) views were used to determine the 80 replicas' thickness at measurement points corresponding to those of the LMMs (ma-b, ma-d, ax-b, inc).

### Accordance between measurements at both sides of each sectional cut (LMMs)

The accordance between the two measurements at both sides of each sectional cut (LMMs) was checked. For this purpose, the mean, standard deviation, and minimum and maximum values of the difference (absolute value) between those measurements were calculated exemplarily for the first measurement of the first investigator [Investigation 1.1 (I1.1)] (Figure 1).

### Intra-rater reliability and agreement (LMMs)

The first investigator repeated the examination of all existing 80 silicone replicas for the LMMs with the frequently used interval of one week in intra-rater reliability studies^1^ ^,^ ^3^ ^,^ ^8^, resulting in two measurements: I1.1 and I1.2. The measurements of the 80 replicas' thickness were analyzed separately for the first and second examination and for the measurement points. The mean, median, minimum, and maximum values were calculated and presented in bar charts. For determining intra-rater reliability, the intraclass correlation coefficient (ICC) was calculated using a two-way random effects model^27^ with the unjustified model ("absolute agreement"). In addition, Bland- Altman bias analyses^2^ were performed to determine the intra-rater agreement. The mean of the differences (bias) between the two measurements (I1.1 minus I1.2; no absolute values) at a specific measurement point was calculated. The closer to zero the mean is, the lower is the bias and the higher the agreement between the two measurements.

### Inter-rater reliability and agreement (LMMs and CASMs)

The measurements were performed by two investigators (postgraduates) with the same professional status (Dr. med. dent. candidates). The training time for the LMMs and ODKMs before starting the measurements was the same for each investigator. The first investigator performed both LMMs (I1.1 and I1.2, twice) and the CASMs (I1, once). For calculation of inter-rater reliability and agreement, only one measurement of the first investigator (LMM) was randomly chosen (I1.2). The second investigator repeated the measurements independently from the first investigator, once for both measurement systems (LMM: I2.1 and CASM: I2) (Figure 1). The measurements of the 80 replicas' thickness were analyzed separately for the first and second investigator for both measurement techniques and for the measurement points. The mean, median, minimum, and maximum values were calculated and presented in bar charts. To quantify the inter-rater reliability, the intraclass correlation coefficient (ICC) was calculated using a two-way random effects model^27^ with the unjustified model ("absolute agreement"). This correlation coefficient is suitable for both intrarater and inter-rater reliability^23^. In addition, Bland- Altman bias analyses^2^ were performed to determine the inter-rater agreement. The mean of the differences (bias) between the two examiners' measurements (LMMs: I1.2 minus I2.1; CASMs: I1 minus I2; no absolute values) at a specific measurement point was calculated.

The IBM SPSS Statistics software (IBM SPSS 21.0; IBM Corporation, Armonk, NY, USA) was used for all analyses.

## Results

### Accordance between measurements at both sides of each sectional cut (LMMs)

The mean ± SD (minimum value, maximum value) of the difference (absolute value) between the two measurements at each sectional cut was 5±7 μm (0, 50 μm) for the marginal values (ma-b, ma-d), 7±6 μm (0, 20 μm) for the axial values (ax-b), and 20±29 μm (0, 190 μm) for the incisal values (inc).

### Intra-rater reliability and agreement (LMMs)

**Figure 3:**
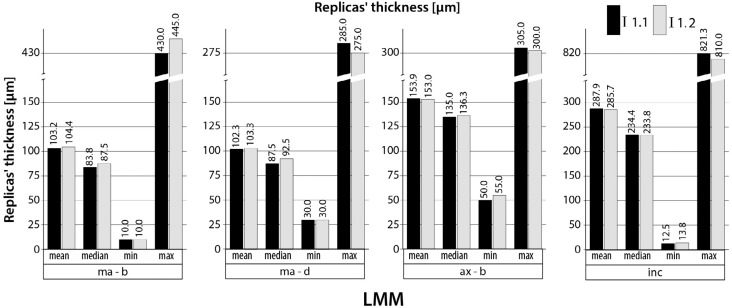
Replicas' thickness (n=80) for the light microscopy measurements (LMMs); I1.1 and I1.2= first and second measurements of the first investigator (LMM); Measurement points: marginal-buccal (ma-b), marginal-distal (ma-d), axial-buccal (ax-b), incisal (inc)

The results of the 80 replicas' thickness measurements (mean, median, minimum, and maximum values) are shown in bar charts (Figure 3) for each examination of the first investigator (I1.1 and I1.2) and for each measurement point. The ICCs for the intra-rater reliability ranged from 0.970 to 0.998 (LMM) and the bias from −1.3 to 2.3 μm, depending on the respective measurement point. For the marginal measurement points (ma-b and ma-d), the first investigator measured the replicas' thickness systematically slightly lower the first time (I1.1) than the second time (I1.2), resulting in a negative bias (e.g. −1.0 μm for ma-d measurement point). For the ax-b and inc measurement points, the first investigator measured the replicas' thickness systematically slightly higher the first time (I1.1) than the second time (I1.2), resulting in a positive bias (e.g. ax-b: 0.9 μm). The ICC and its 95% confidence interval (CI), as well as the bias are shown in Table 1 for each specific measurement point.

**Table 1:** Intraclass correlation coefficient analyses for intra-rater reliability for LMM and for inter-rater reliability for LMM versus CASM; Bland-Altman bias analysis for intra-rater agreement for LMM and for inter-rater agreement for LMM versus CASM

	ICC (LL; UL) for intra-rater reliability (LMMs; I1.1 and I1.2)	ICC (LL; UL) for inter-rater reliability (LMMs; I1.2 and I2.1)	ICC (LL; UL) for inter-rater reliability (CASMs; I1 and I2)	bias [μm] for intra-rater agreement (LMMs; I1.1-I1.2)	bias [μm] for inter-rater agreement (LMMs; I1.2-I2.1)	bias [μm] for inter-rater agreement (CASMs; I1-I2)
ma-b	0.993 (0.990;0.996)	0.978 (0.958;0.988)	0.984 (0.975;0.990)	-1.3	-7.6	0.8
ma-d	0.970 (0.954;0.981)	0.848 (0.702;0.915)	0.994 (0.991;0.996)	-1.0	-15.7	1.9
ax-b	0.992 (0.988;0.995)	0.984 (0.974;0.990)	0.945 (0.916;0.965)	0.9	3.5	-3.0
inc	0.998 (0.998;0.999)	0.998 (0.997;0.999)	0.999 (0.999;1.000)	2.3	-2.2	-1.4

Definitions: ICC= intraclass correlation coefficient; LL= lower limit; UL= upper limit; LMMs= light microscopy measurements; CASMs= computer-assisted measurements; I1.1 and I1.2= first and second measurements of the first investigator (LMM); I2.1= measurement of the second investigator (LMM); I1 and I2= measurements of the first and second investigators (CASM); bias= mean of differences between measurements at specific measurement points.

Measurement points: marginal-buccal (ma-b), marginal-distal (ma-d), axial-buccal (ax-b), incisal (inc).

### Inter-rater reliability and agreement (LMMs and CASMs)

**Figure 4:**
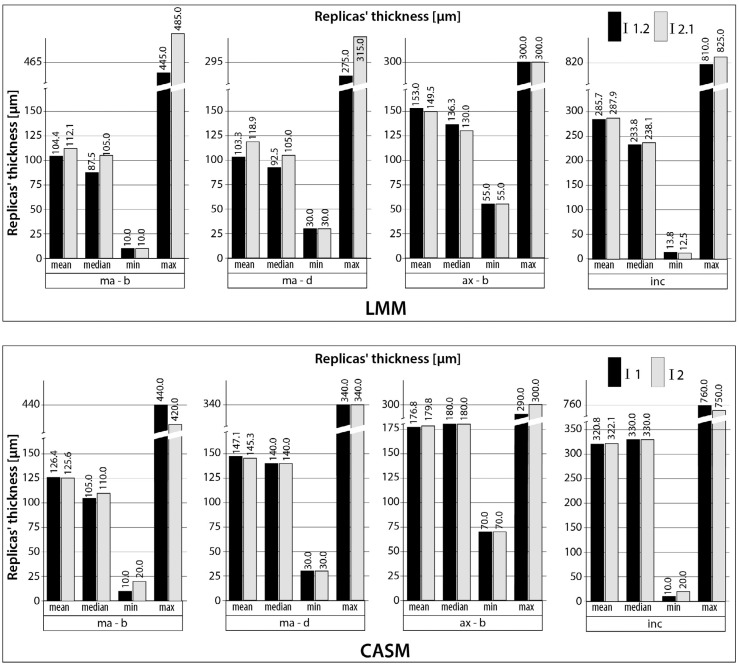
Replicas' thickness (n=80 for each measurement technique) for the light microscopy measurements (LMMs) and for the computer-assisted measurements (CASMs); I1.2= second measurements of the first investigator (LMM); I2.1= measurement of the second investigator (LMM); I1 and I2= measurements of the first and second investigators (CASM); Measurement points: marginal-buccal (ma-b), marginal-distal (ma-d), axial-buccal (ax-b), incisal (inc)

The results of the 80 replicas' thickness measurements (mean, median, minimum, and maximum values) are shown in bar charts (Figure 4) for each examination (LMM: I1.2 versus I2.1; CASM: I1 versus I2) and for each measurement point. The ICCs for the inter-rater reliability ranged from 0.848 to 0.998 (LMM) and from 0.945 to 0.999 (CASM). The bias also showed a wider range between the specific measurement points for the LMMs (-15.7 to 3.5 μm) than for the CASMs (-3.0 to 1.9 μm). For the LMMs, the first investigator (I1.2) measured the replicas' thickness systematically lower (exception: measurement point ax-b) than the second investigator (I2.1), resulting in a negative bias (e.g. −15.7 μm for ma-d measurement point). The ICC and its 95% CI and the bias are shown in Table 1 for each measurement point.

## Discussion

The ICC for the intra-rater reliability of the LMMs shows very high values and a rather narrow 95% CI. The bias shows values very close to zero for all measurement points. These results indicate high repeatability of LMMs in the current study, regardless of the specific measurement points. Thus, the study's first hypothesis can be accepted, and the third hypothesis can also be accepted for the intra-rater evaluation of the LMMs.

The inter-rater reliability for the LMMs shows similarly high values for the ICC, except for the ma-d measurement, which has a slightly lower ICC (<0.9) and a wider 95% CI for the ICC. The bias shows this dependence on the measurement point more clearly. Although the bias for the ax-d and inc measurement points is still close to zero, the ma-b bias is almost 6 times higher and the ma-d bias is almost 16 times higher than the bias for the intra-rater agreement. The first investigator systematically determined lower marginal values (ma-b, ma-d) than the second investigator, which may have been due to the difficulty identifying the exact marginal measurement point. The light-body material thins toward the margins, in contrast to the thicker layers that are found axially and incisally. Thus, the Bland-Altman bias gave a more differentiated view than the ICC, as has been previously claimed^2^. In summary, the results indicate a subjective influence for the LMMs at the ma-b and ma-d measurement points. The inter-rater reliability of the LMMs still seems acceptable, however, given the clinically acceptable marginal fit values^7^ ^,^ ^29^. Thus, the study's second hypothesis can be accepted, whereas the third hypothesis must be rejected for inter-rater evaluation using LMMs.

The inter-rater reliability for the CASMs shows very high ICC values with a rather narrow 95% CI. The bias values are closer to zero than the LMMs' bias values for every measurement point and are rather independent of the measurement point. These differences can be explained by the different method of determining the replica's thickness. For the CASM technique, no perpendicular measurement^5^ is needed. Instead, the thickness is automatically given in the color-coded difference images (needle plot) at a specific measurement point determined by the examiner. In summary, the results indicate that the CASM technique is somewhat more objective than the LMM technique and is independent of the measurement point. Thus, the study's second and third hypotheses can be accepted for the CASM technique.

The influence of the investigators' professional status on the measurement results was not evaluated, which could be considered a limitation of the study. However, for the inter-rater analyses, the measurements were performed by two investigators (postgraduates) with the same professional status (Dr. med. dent. candidates). Thus, a comparison between two individuals' measurements (inter-rater) was possible without a further interfering factor (professional status). However, the influence of this possible interfering factor is a further aspect of interest and should be addressed in future studies in this research field.

The use of computer-assisted analyses is a constantly growing field not only in dental research. While the conventional replica technique does not require high investment costs and is easily learned, the opposite is true for the CASM technique. The latter technique implies the use of complex software for digitizing and analysis. Fortunately, topical software are becoming more user-friendly.

As an *in vitro* investigation, this study shows the well-known limitations of *in vitro* studies compared to *in vivo* studies; e.g. it is lacking a randomized design in contrast to a randomized, controlled clinical trial (RCT). A specific limitation of *in vitro* coping fit analyses studies is the artificially superior coping fit compared to the achievable coping fit under *in vivo* conditions. Clinical factors, e.g. saliva or blood contamination, during dental impression making or margin geometry, lead to unintended modifications in coping fit^18^. In addition, *in vivo* studies evaluating the intra- and interrater reliability of measurement techniques usually include different patients^1^ ^,^ ^3^ ^,^ ^8^. To simulate *in vivo* conditions, we performed modifications on purpose to create different CAD models. The different CAD models successfully resulted in copings with varying fit and thus varying replicas' thickness between the samples as shown before^14^, which can also be seen in the wide range of values for the replicas' thickness for each measurement point (Figures 3 and 4). A further positive aspect of this *in vitro* study is the measurement of 80 replicas resulting in a rather high sample size. This enhances the quality of our analyses.

The analyses of the 80 replicas' thickness showed similar values (mean, median, minimum, and maximum values) for both measurements of the first investigator (intra-rater testing). For the inter-rater testing, these values showed higher accordance between both investigators' measurements for the CASM than for the LMM, which is in accordance with the results of the ICC and bias analyses above. The CASMs tended to show higher mean values for the replicas' thickness than the LMMs. This phenomenon has already been described in a study that compared both techniques^14^. The difference between the values is in the range of powder thickness (20-40 μm under ideal conditions)^4^ ^,^ ^20^. For the CASM technique, a powder had to be applied on the replica against surface reflection and light scattering. Using optically digitizable silicones would eliminate the need for the powder^20^. However, this phenomenon did not influence the CASM inter-rater analyses as both examiners performed the measurements on the same, previously powdered samples.

For the LMMs, we used the mean at both sides of each sectional cut for the marginal and axial values and the mean of eight measurements (both sides of four sectional cuts) for the incisal value. This was done to have points analogous to those used for the CASMs, in which no cutting took place, and to reduce error^15^. To estimate the impact of this procedure, we determined the accordance between the measurements at both sides of each sectional cut. This analysis revealed a rather high accordance for the marginal and axial values. The accordance for the incisal values was lower but still clinically sufficient.

For the CASM technique, the replicas were manufactured on duplicate plaster dies due to the stainless steel master dies' reflection. For the LMM technique, the replicas were directly manufactured on the master die. The mean values for the difference between duplicate plaster dies and steel master die are +9.2/-8.5 μm (SD 1.1/0.5) for the identical plaster dies' manufacturing procedure (identical master die/ impression technique and material/ plaster material)^24^. Thus, areas of reduction (mean: −8.5 μm) and areas of enlargement (mean: +9.2 μm) compared to the steel master die were found resulting in higher or lower thickness of the replicas on the plaster dies for the CASMs. However, this error can be classified as negligible, as the mean values are in the range of the measurement uncertainty of 8 μm of the ODMK 97 digitizing system, and show a very low standard deviation (SD 1.1/0.5)^24^. Besides, the error can be classified as a systematic one due to the identical manufacturing procedure for all gypsum dies. It did not influence the CASM inter-rater analyses, as both examiners performed the measurements on the same samples.

Despite multiple studies evaluating the crown fit with the replica technique using LMMs^22^, their intra- and inter-rater reliability and agreement has rarely been determined. Molin and Karlsson^21^ (1993) determined the mean difference and the coefficient of correlation between pairwise measurements for the replica technique with LMMs for inlay preparations. However, they did not specify how they calculated the mean difference (absolute values or not: "bias") and the coefficient of correlation. They also did not clarify whether the pairwise measurements had been performed by a single investigator (intra-rater reliability) or different investigators (inter-rater reliability). Neither the measurement points nor the inlay material used for the correlation analyses was specified. Thus, our study is the first to have evaluated both intra- and inter-rater reliability (ICC) and agreement (Bland-Altman bias) for the replica technique with LMMs separately for different measurement points. The replica technique with CASMs^14^ ^,^ ^20^ used in this study had not been previously evaluated for its reliability.

The same two investigators performed both techniques, LMMs and CASMs, using identical copings. Thus, for the first time, a direct comparison of interrater reliability and agreement between the LMM and CASM techniques was possible, which adds new information to studies in this area. The comparison showed slightly higher reliability and agreement with the CASM technique and no dependence on the measurement points — in contrast to the LMM technique.

Light microscopy and the computer-assisted replica technique are used in studies to evaluate new materials used and new manufacturing procedures applied for dental restorations for daily clinical practice^9^ ^-^ ^14^ ^,^ ^20^ ^,^ ^21^ ^,^ ^30^. Given the clinically acceptable marginal fit values^7^ ^,^ ^29^, both techniques are reliable methods for the evaluation of dental restoration fit prior to the clinical use of new materials and manufacturing procedures for dental restorations. However, the CASM technique shows slightly superior reliability, especially for determining the marginal fit.

## Conclusion

The following conclusions can be drawn for the internal replica technique for evaluating the marginal and internal fit of dental copings.

The light microscopy replica measurements showed high intra-rater reliability and agreement (repeatability) and somewhat worse, but still clinically acceptable, inter-rater reliability and agreement at the marginal measurement points.

The computer-assisted replica measurement was slightly more objective than the light microscopy replica measurement and was independent from the measurement point.
